# Placental Pathology in COVID-19: Case Series in a Community Hospital Setting

**DOI:** 10.7759/cureus.12522

**Published:** 2021-01-06

**Authors:** Natasha Singh, Tinera Buckley, Wendy Shertz

**Affiliations:** 1 Pathology, Robert Wood Johnson (RWJ) Barnabas Health, Livingston, USA; 2 Pathology, Monmouth Medical Center, Long Branch, USA

**Keywords:** placental pathology, obstetrics, infectious disease, microscopic features, covid-19, malperfusion, microcalcifications, fibrin thrombi, perinatal pathology, maternal medicine

## Abstract

Objective

To report the histopathologic findings in the placentas of pregnant women with coronavirus disease-19 (COVID-19).

Methods

Pregnant women with COVID-19 delivering between April 2020 to June 2020 were identified. A retrospective study of placentas from COVID positive women received in the Department of Pathology, Monmouth Medical Center affiliate of Robert Wood Johnson Barnabas Health were examined and compared to control cohort of placentas from COVID negative women. The mothers were tested for coronavirus through nasopharyngeal swab upon admission to labor and delivery. The placentas from mothers who tested negative for the virus were sent to Pathology for examination based on the obstetrician’s clinical judgment.

Results

Fifty surgical specimens (49 placentas and one product of conception) from patients positive for COVID-19 were examined and compared with fifty placentas from women with negative COVID-19 test results, who delivered during the same period. Most of the neonates had Appearance, Pulse, Grimace, Activity and Respiration (APGAR) scores of 9 and 9 at 1 and 5 minutes, respectively. Increased incidence of the COVID-19 positivity was noted in individuals with Rh-positive blood group A and Jewish heritage. Compared to the control group, the COVID-19 positive placentas showed increased features of malperfusion (microcalcifications, fibrin thrombi, syncytial knotting, and villous agglutination). However, there was no significant dysregulation in other variables, such as inflammation or coagulation. There was no case of maternal or fetal death (greater than eight weeks) or evidence of worse fetal outcomes noted due to a mother's positive COVID-19 status.

Conclusions

The COVID-19 positive placentas showed an increased prevalence of microcalcifications and fibrin thrombi, which may reflect an underlying hypercoagulable state induced by COVID-19 infection or could be due to excessive syncytiotrophoblast injury.

## Introduction

The novel viral respiratory disease caused by severe acute respiratory syndrome coronavirus 2 (SARS-CoV-2), is responsible for the pandemic of the coronavirus disease-19 (COVID-19) cases worldwide [1]. Coronavirus is a positive single-stranded enveloped RNA virus belonging to the family Coronaviridae [2]. The term Coronaviridae is derived from the structural characteristic of the crown-like or halo-like appearance of the glycoprotein. COVID-19 was first detected in Wuhan, China [2]. Originally, bats were the only known hosts for this virus [1]. It is known to affect the gastrointestinal and respiratory systems of its host primarily and is mainly found in the avian and mammalian species. The route of transmission is airborne droplets, which enter the respiratory system via the nasopharynx. The four most common human coronaviruses known are - 229E, NL63, OC43, and HKU1. Other viruses that belong to the same family are the Middle East respiratory syndrome (MERS) and severe acute respiratory syndrome (SARS) viruses [1].

So far, the impact of the COVID-19 on pregnant women and infants is largely unknown and is a key area of interest. A comparison of the histopathological examination of the placenta from COVID-19 positive and COVID-19 negative mothers can provide significant information regarding the coronavirus's effect on maternal and fetal outcomes. Several viral infections are associated with specific pathological findings, which vary from active processes within various cells to vascular changes, including inflammatory and obliterative lesions, ultimately leading to fibrosis of the villous stroma [3]. For example, there have been case reports of intervillositis in patients infected with Zika and Dengue virus [4]. Researchers in China have documented placental histopathological findings in COVID-19 positive patients to include increased perivillous fibrin deposition, villous infarcts, and chorioangioma [5]. The current study presents new histopathological findings in the placenta of COVID-19 positive patients.

## Materials and methods

Pregnant women with COVID-19 delivering between April 01, 2020 to June 30, 2020 were identified via the electronic health record. The study presents findings in 100 women, 50 COVID-19 test-positive patients, and 50 COVID-19 test negative control patients. All tissue specimens were received at the Department of Pathology at Monmouth Medical Center, New Jersey, and studied retrospectively. Placentas were grossly examined and evaluated via hematoxylin and eosin staining and compared to controls of women with negative COVID-19 test. The mothers were tested for coronavirus through nasopharyngeal swab upon admission to labor and delivery. The placenta and newborns were not tested for COVID-19. All the placentas from COVID-19 positive mothers were sent to the Department of Pathology. The placentas from mothers who tested negative for the virus were sent to Pathology for examination based on the obstetrician’s clinical judgment.

## Results

One hundred surgical specimens were examined from patients tested for COVID-19 (SARS-CoV-2) over a three-month period. Forty-nine placentas and one product of conception were received from patients testing positive for COVID-19 and fifty placentas were received from patients testing negative for COVID-19. Two placentas were classified as twin placenta (1=diamniotic dichorionic, 1= monoamniotic dichorionic). Only one COVID-19 positive patient had documented symptoms of loss of taste and smell, which had occurred one month prior to delivery. The remaining viral positive patients did not report having previous or current symptoms. The most common comorbidities identified in both cohorts were gestational diabetes mellitus, thyroid abnormalities, and hypertension +/- pre-eclampsia. There were two patients with hemoglobinopathies (i.e. sickle cell anemia and hemophilia A) in the viral positive cohort. One unique demographic finding was the increased number of Jewish patients in the viral positive cohort (61%) compared to 30% observed in the viral negative control population (Table 1). This finding was unexpected as there were no inclusion or exclusion criteria used to determine the participants in either group. 

**Table 1:** Comparison of demographic and clinical features of COVID-19 patients vs. controls WGA= weeks gestational age; GA= gestational age; GBS= group B strep; GDM= gestational diabetes mellitus; GHTN= gestational hypertension; LFT= liver function test; NSVD= normal spontaneous vaginal delivery; PLT= platelets.

	Avg age (yrs)	Avg GA (wga)	Clinical factors	Jewish heritage	Most common blood type	Coagulation abnormalities
COVID-19 patients	29	40.3	NSVD (.92)	.61	O (.25)	Platelets (.12)
			GBS+ (.24)		A (.37)	LFT (.06)
			GHTN (.02)		B (.24)	Fibrinogen (.02)
			Thyroid abnormalities (.02)		AB (.09)	PT/PTT (.02)
			GDM (.02)			
			Other (.04)			
Controls	33	38.2	NSVD (.24)	.30	O (.44)	LFT (.30)
			GBS+ (.26)		A (.34)	Platelets (.12)
			GHTN (.06)		B (.16)	PT/PTT (.08)
			Thyroid abnormalities (.08)		AB (.06)	Fibrinogen (.06)
			GDM (.12)			
			Other (.10)			

The two groups were comparable in age (range from 17-42, average 31). The gestational ages at delivery were third trimester in both cohorts, with exception of the eight-week point-of-care (POC) and a second trimester premature delivery at 21.6 weeks, both of which were viral positive (Table 1). The maternal/fetal outcomes were all stable and/or asymptomatic at discharge and all of the deliveries resulted in viable live births (male=25, female=26), except the products of conception specimen, which was delivered by a COVID-19 positive patient after a spontaneous abortion at eight weeks gestation. However, laboratory testing showed that the viral negative patients had a slightly greater, but not significant, occurrence of perinatal coagulation abnormalities compared to the COVID-19 positive mothers (Table 1). In addition, normal spontaneous vaginal deliveries without complications occurred more often in the COVID-19 positive cohort at rates of up to 94%, compared to 76% observed amongst mothers in the control population, who had a higher incidence of cesarean section delivery at this facility (Table 1). However, the viral positive patients had increased occurrence of type A blood group (37%), compared to increased type O blood group observed in the viral negative cohort (Table 1). 

**Figure 1:**
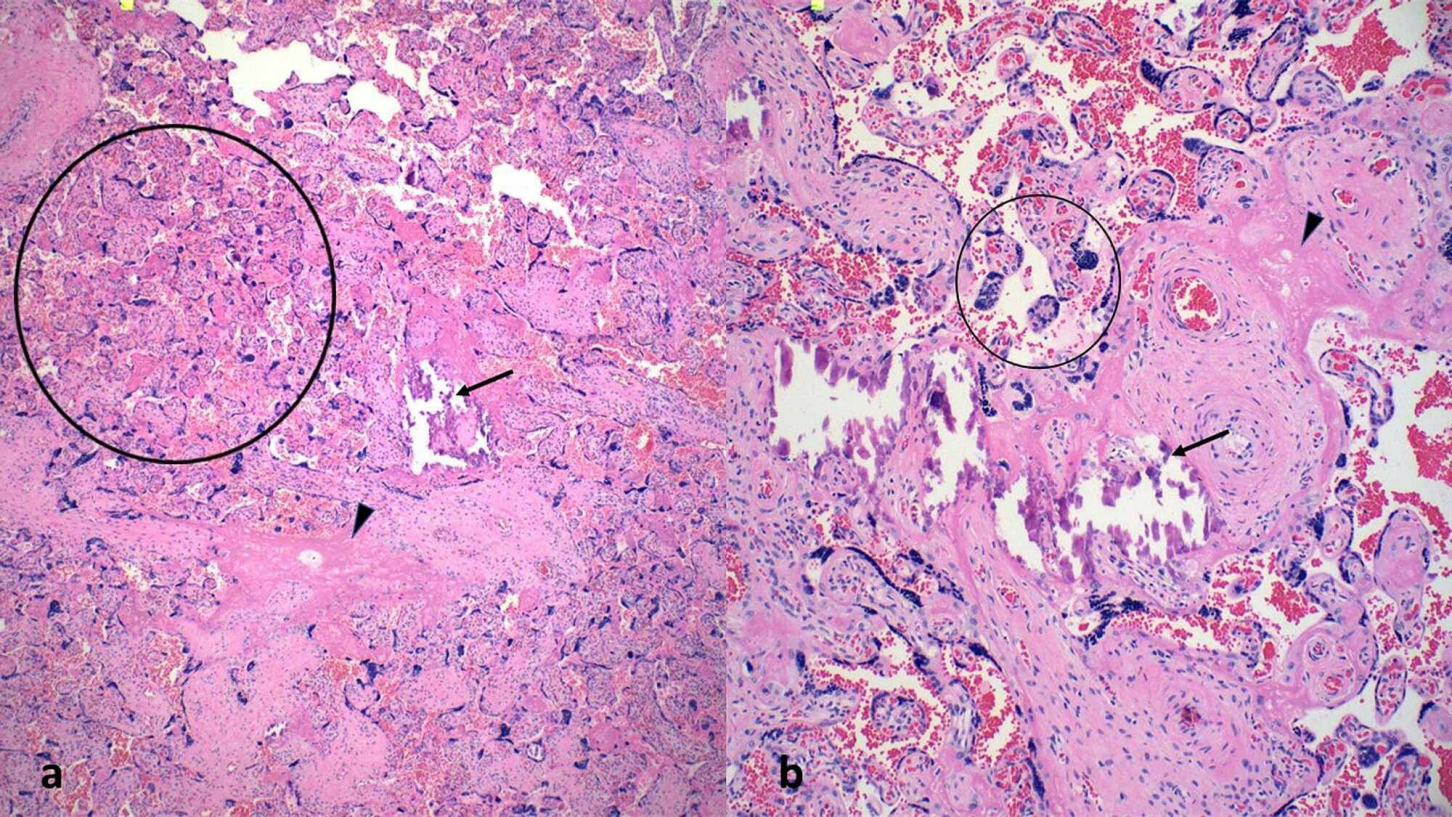
Microscopic findings in viral positive placenta Images a (10x) and b (20x) show the microscopic findings observed in COVID positive placentas including increased microcalcifications (arrow), increased fibrin (arrowhead), and increased syncytial knotting (circled).

Microscopic examination of all 100 specimens revealed findings seen in 46 of the 50 viral positive specimens, which included increased microcalcifications, increased fibrin, increased syncytial knotting, small fibrotic villi, and villous agglutination (Figure 1).

These findings were present in varying degrees but were consistently observed in the placentas and the one product of conception. Comparatively, similar evidence of maternal malperfusion was seen in only 14% of the viral negative patients. Other microscopic findings in both populations included no histopathologic abnormality, chorangiosis, abruption, chorioamnionitis/funisitis, and meconium staining (Table 2).

**Table 2:** Comparison of microscopic features of COVID-19 patients vs controls Bold P values indicate significant results. Data are given as No. (%) MVM= maternal vascular malperfusion.

	COVID-19 patients (n=50)	Controls (n=50)	Odds ratio vs control	P value vs Controls (p< .05)
Features of maternal/fetal malperfusion				
MVM- formal diagnosis	0 (0)	0 (0)	-	-
Increased fibrin thrombi/focal infarction	13 (26)	6 (12)	2.58	.075
Increased fibrin	26 (52)	3 (6)	17	< .01>
Increased microcalcifications	30 (60)	2 (4)	35	< .01>
Small fibrotic villi	14 (28)	0 (0)	0	< .01>
Villous agglutination	9 (18)	0 (0)	0	< .01>
Increased syncytial knotting	20 (40)	1 (2)	32.8	< .01>
Chorangiosis	7 (14)	2 (4)	3.89	.08
Other placental findings				
No histopathological abnormalities	5 (10)	28 56)	.09	< .01>
Meconium staining	3 (6)	7 (14)	.39	.18
Chorioamnionitis/Funisitis	3 (6)	8 (16)	.33	.11

The latter was often associated with clinical presentations of preterm contractions and premature/prolonged rupture of membranes. However, the most common microscopic finding in the viral negative cohort was third trimester placenta, membranes and cord without histopathological abnormalities (56%). Gross placental examination did not reveal any distinct findings associated with COVID-19 status.

## Discussion

Coronavirus is a family of viruses known to cause a wide range of respiratory symptoms [6]. In the last few years, three new coronaviruses with animal hosts have led to severe complications in human beings. These three coronaviruses include MERS, SARS, and SARS-CoV-2 [7,8]. In 2003, the SARS-CoV1 virus had emerged in Guangdong, China, and had spread to 26 different countries but was not a global pandemic like the SARS-CoV21. SARS-CoV1 has been associated with thrombotic complications [9]. There had been reports in the past regarding histopathological findings compatible with edema and fibrin thrombi in the pulmonary vasculature [2,10]. There is sparse data available regarding coronavirus's effect on the placenta, maternal, and fetal outcomes [2]. Studies in the past have suggested that SARS, which emerged in China in 2004, was associated with increased microcalcifications in viral positive mothers' placentas [7,9]. In this study, an attempt was made to gather cases from confirmed viral positive mothers and follow up with their outcomes after delivery as well as fetal outcomes and placental pathology.

The striking similarity between placental pathology in SARS-CoV1 and SARS-CoV-2 has been increased fibrin, which could be caused by disturbances in maternal placenta blood flow due to the low oxygen level from the hypoxic respiratory disease and the presence of microcalcifications [3]. It is presently unknown whether microcalcifications are a sign of viral infection by the coronavirus family or if they have a completely different etiology. COVID-19 positive specimens show increased intervillous thrombi [11]. It is known that the intervillous space mainly contains maternal blood; however, 85% of the villous circulation is suggested to be of fetal origin. Other pregnancy-related conditions, such as maternal hypertension, have been documented to promote thrombi formation [2,4,5,12-13]. Therefore, pregnancy promotes substantial hemodynamic and prothrombotic change, potentially leading to vascular complications during pregnancy and in the post-partum period. We evaluated these placentas for features like thrombi, microcalcifications, increased syncytial knotting, among others. There were increased microcalcifications and thrombi found in placentas, which might be related to disturbances in maternal placental blood flow due to the hypoxic respiratory disorder. Studies in the past have also suggested the dysregulation of coagulation cascade attributed to the coronavirus leading to fibrin clot formation [1,2,9,12-13]. Increased syncytial knotting is a microscopic finding that could be a result of uteroplacental malperfusion. Our study also includes a product of conception specimen resulting from an intrauterine fetal death delivered by a COVID-19 positive mother. This raises the possibility of that COVID-19 causes adverse effects on the fetus in utero, which is an area that deserves further investigation. There have been few studies published recently which focus on placental findings in COVID-19, but none of these studies describe increased prevalence of microcalcifications and focus on such a large sample size. The main highlights of these studies have been the increased prevalence of decidual arteriopathy and other features of maternal vascular malperfusion [1,2,9]. Our study focuses on an increased prevalence of microcalcifications and fibrin thrombi as compared to negative controls. Microscopic examination of 90% of the viral positive placentas revealed increased microcalcifications and fibrin thrombi.

All the findings in this community case series indicate that COVID-19 could be related to hypercoagulability conditions resulting in uteroplacental malperfusion. The study focuses on initial findings in the COVID-19 placenta, which can provide insight into the impact of this virus on maternal and fetal health. COVID-19 has been associated with hypercoagulability, pulmonary emboli, and increased D-dimer levels [12,13]. The microscopic findings of increased fibrin deposition can be related to hypercoagulability or other etiology and require further studies. It is notable that mothers in the current study did not experience complications that warranted monitoring of D-dimer levels although coagulopathy abnormalities were slightly more common in the viral negative patients (Table 1). However, there were no significant hematology abnormalities observed during or near delivery that resulted in adverse outcomes in either population. One major limitation of this study involves the lack of long-term maternal and fetal follow-up, and further studies to determine long-term effects. However, this article represents the largest comparison of COVID-19 positive versus COVID-19 negative placentas in a community hospital setting. 

## Conclusions

SARS-CoV-2 is the causative agent of COVID-19, which has resulted in the current global pandemic. No prior study has assessed a large population size to discuss placental pathology found in COVID-19 positive individuals in a community-based hospital setting. The COVID-19 positive placentas showed an increased prevalence of microcalcifications and fibrin thrombi, which may reflect an underlying hypercoagulable state induced by COVID-19 infection, or due to excessive syncytiotrophoblast injury. In our study, there is no evidence of worse fetal or neonatal outcomes due to the positive COVID-19 status of the mother. However, the long-term effects of COVID-19 infection on mother and infant health warrant further study.
